# Recent advances in understanding and managing pediatric nonalcoholic fatty liver disease

**DOI:** 10.12688/f1000research.24198.1

**Published:** 2020-05-19

**Authors:** Jennifer Vittorio, Joel E. Lavine

**Affiliations:** 1 1. Division of Pediatric Gastroenterology, Hepatology and Nutrition, Columbia University Vagelos College of Physicians and Surgeons, New York, USA

**Keywords:** Nonalcoholic Steatohepatitis, Pediatric Fatty Liver Disease, Obesity, Noninvasive Biomarkers, Therapeutics

## Abstract

Nonalcoholic fatty liver disease (NAFLD) represents a spectrum of disease that can range from isolated macrovesicular hepatocellular steatosis to nonalcoholic steatohepatitis (NASH) with or without fibrosis to cirrhosis. The prevalence of NAFLD has increased over several decades, mirroring the global obesity pandemic. NAFLD currently represents the most common etiology of chronic liver disease in children and adolescents worldwide. Disease presentation in childhood strongly suggests that these children may have unique susceptibilities and more severe long-term consequences. Emerging data demonstrate that the pathogenesis of early-onset NAFLD is secondary to a complex interplay involving genetic, metabolic, environmental, and microbiological factors. Such influences may begin *in utero*. Dietary and lifestyle modifications remain the primary effective therapeutic interventions, although long-term efficacy is limited by poor adoption or adherence. Advances in the development and validation of non-invasive biomarkers and imaging modalities will facilitate diagnosis for affected children and adolescents and facilitate long-term natural history studies and the development of therapeutic interventions.

## Introduction

Nonalcoholic fatty liver disease (NAFLD) represents a clinical spectrum of liver abnormalities resulting from excessive fat accumulation within hepatocytes. These abnormalities may range from simple steatosis to nonalcoholic steatohepatitis (NASH) with evident inflammation and cell injury. In some, NASH progresses to advanced fibrosis, cirrhosis, or increased risk for hepatocellular carcinoma. The prevalence of NAFLD has increased over the last several decades in tandem with the rise in obesity globally. Currently, NAFLD represents the most common etiology of chronic liver disease in children and adolescents, affecting nearly 10% of the pediatric population in some parts of the developed world ^1, 2^. Over the last decade, it has become a leading indication for liver transplantation in adults ^3^, mandating the need for early recognition and treatment.

Earlier-onset NASH may result from a distinct underlying pathogenesis, which is demonstrable by differences in certain features relating to histopathology, evident in a subset of prepubertal children ^4^. Emerging data support the idea that individuals whose NAFLD is diagnosed in childhood have an increased risk of morbidity and mortality in adulthood ^5^. These findings show that affected children may be afflicted with a more aggressive disease phenotype. It follows that, when NAFLD presents earlier, these children are at risk for a more advanced liver disease entering adulthood. Also, they may be at heightened risk for other comorbidities related to metabolic syndrome, including cardiovascular disease.

Some unique pediatric histologic features include a more pronounced fat accumulation, increased portal inflammation and fibrosis, and a less pronounced lobular inflammation and ballooning degeneration. Earlier presentation of disease, along with discrepant features of histology, suggests that children and adults may differ in regard to pathophysiology, and children may display enhanced genetic and environmental vulnerability. Some *in utero* and perinatal stressors such as premature birth may predispose pediatric patients to NAFLD ^6^. Recently, our understanding of NAFLD has improved significantly with advances in diagnosis, etiopathogenesis, genetic predispositions, and intervention.

## Epidemiology

The prevalence of pediatric NAFLD is difficult to ascertain since most studies use indirect indicators of hepatic steatosis. Furthermore, the definitive diagnosis of NASH requires a histologic diagnosis via liver biopsy, which is not feasible in population-based studies. With indirect measures, including ultrasound-based imaging or elevated serum aminotransaminase values, the prevalence of NAFLD has been reported as 7% among children and adolescents in the general population and upwards of 34% among obese children ^1^. When autopsy studies from Poland, Turkey, and the United States are considered, the worldwide prevalence of NAFLD ranges from 4.2 to 9.6% and increases to 38% in those with obesity ^2, 7– 9^. Hispanic children, particularly of Mexican descent, have the highest incidence of NAFLD when body mass index (BMI) z-score, gender, and age are controlled for, whereas black children, who have the lowest reported incidence, seem to be relatively protected from the accumulation of hepatic fat (HF) ^2, 10^. There is a male predominance in pediatric studies and the problem is most often detected in the early pubertal period ^11^.

It is important to recognize that current prevalence studies likely do not reflect the true prevalence of disease given the lack of uniform screening methodologies and consistency in diagnostic criteria. Data on the prevalence of NASH, as opposed to NAFLD without NASH, require autopsy-based studies of liver histology in children and adolescents dying from non-medical causes.

## Pathophysiology

The clinical spectrum of NAFLD results from specific lipotoxicity (or lipotoxicities) with or without augmented oxidative stress that is found in connection with chronic excess of hepatic lipids. Hepatocellular steatosis in some provides the substrate for invoking hepatic inflammation that predisposes patients to cell injury, apoptosis, stellate cell activation, and production of excess matrix. Why certain individuals develop progressive disease whereas others are less affected remains less clear. Although the majority of children with NAFLD are obese, not all children with obesity develop NAFLD. NAFLD can occur, albeit much less often, in lean individuals. In these cases, further evaluation is especially crucial to assess for alternative etiologies. Several conditions, such as Wilson’s disease, appear similar to NAFLD and may have available therapies ^12, 13^.

### Risk factors

Clear risk factors for NAFLD include male gender, obesity, and increased waist circumference ^14, 15^. In an epidemiologic study, increased fructose intake was associated with NAFLD in adolescents with obesity ^16^. Fructose promotes increased visceral adiposity, decreased insulin sensitivity, and increased hepatic *de novo* lipogenesis which potentiates intrahepatic fat accumulation ^17, 18^. Visceral obesity and insulin resistance are important risk factors for pediatric NAFLD ^19^. Increasingly, sedentary lifestyles and diminished attention to healthier diets are the primary drivers for the increase in obesity prevalence.

The reported incidence of obstructive sleep apnea (OSA) in pediatric NAFLD is 40 to 60%. Several studies have suggested that chronic intermittent hypoxia secondary to OSA and excess production of reactive oxygen species may contribute to the progression of pediatric NAFLD and increased fibrosis independently of BMI ^20, 21^. Data regarding the effect of treatment with continuous positive airway pressure therapy on NAFLD are mixed. Long-term prospective studies of a larger scale are necessary, particularly in the pediatric population ^22^.

### Genetics

NAFLD appears to have a strong heritable component based on ethnic differences in prevalence and clustering within families independently of adiposity. Schwimmer *et al*. demonstrated that NAFLD was significantly more common in siblings (59%) and parents (78%) of children with NAFLD when compared with siblings (17%) and parents (37%) of BMI-matched children without NAFLD ^23^.

Genetic polymorphisms of patatin-like phospholipase domain–containing protein 3 ( *PNPLA3*), transmembrane 6 superfamily member 2, and glucokinase regulatory protein ( *GCKR*) are associated with the risk of NAFLD in children ^24– 28^.

### Microbiome

There has been an increased recognition regarding the role of gut microbiota and an association with the development and progression of NAFLD. Patients with NASH appear to have intestinal microbiota enriched in alcohol-producing bacteria, such as *Escherichia coli*, and elevated blood ethanol levels have been documented. The abundance of endogenous alcohol-producing bacteria in NASH microbiomes likely contributes to disease pathogenesis as these bacteria provide a constant source of reactive oxygen species which accompanies an increase in intestinal permeability and hepatic inflammation by way of direct delivery of intestinal venous blood to hepatocyte Toll-like receptors (TLRs) via the portal vein ^29^.

In a recent study of children with obesity or NAFLD, the intestinal microbiomes of children with NAFLD were found to have a decreased α-diversity and increased variation in β-diversity, otherwise known as dysbiosis, when compared with obese children without NAFLD ^30^. NAFLD and increased disease severity were also associated with an increased abundance of genes encoding for pro-inflammatory bacterial products ^30^. Alterations in the intestinal microbiome may contribute to the pathogenesis of NAFLD and should be further evaluated as markers of disease and severity.

### Developmental programming: perinatal influence

Many aspects of the mother–child dyad have been examined to identify potentially modifiable risk factors for infant and maternal NAFLD. Maternal obesity, antepartum hyperglycemia, gestational diabetes, excessive weight gain during pregnancy, cesarean section, early-life exposure to antibiotics, and the absence of breastfeeding have all been associated with increased risk for obesity in offspring and the development of NAFLD ^31, 32^.

**Figure 1.**
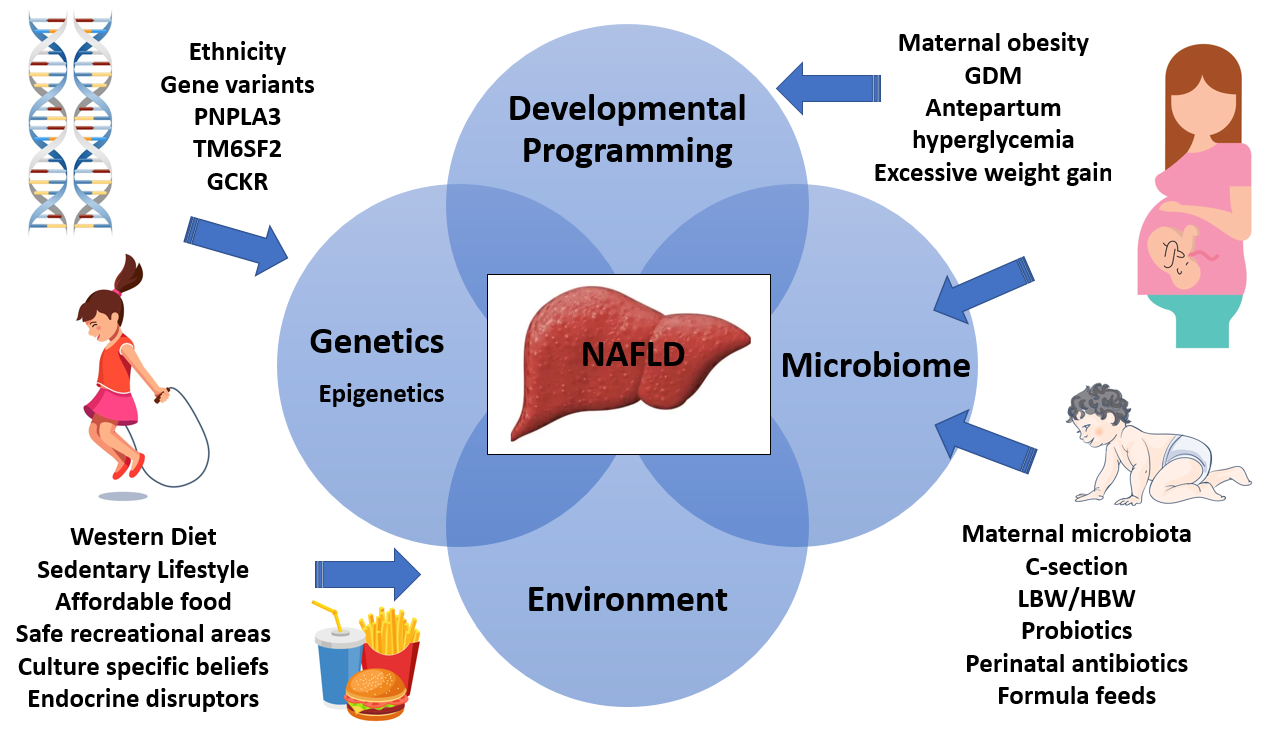
Interplay between genetics, epigenetics, the microbiome, and environmental factors that contribute to NAFLD. GCKR, glucokinase regulatory protein; GDM, gestational diabetes mellitus; HBW, high birth weight; LBW, low birth weight; PNPLA3, patatin-like phospholipase domain–containing protein 3; TM6SF2, transmembrane 6 superfamily member 2.

Altered intestinal microbiomes in the infants of obese mothers result in altered bile acid metabolism, increased short-chain fatty acids, and increased intestinal permeability with bacterial translocation. Dysbiosis also has implications for the bone marrow with recruitment of poorly functioning macrophages. Hepatic inflammation secondary to the translocated bacteria, or their endotoxins which stimulate hepatocyte TLRs triggering a pro-inflammatory cascade, is inadequately controlled because of these hyporesponsive macrophages. A secondary hit, such as that found in Western diets, results in hepatic steatosis and obesity ^33, 34^. See Figure 1 for an illustrative summary of the pathophysiology of NAFLD.

## Screening/diagnosis

There is a current discrepancy in recommendations between societal guidelines in regard to the utility of screening for pediatric NAFLD. In 2017, both the American Association for the Study of Liver Diseases (AASLD) ^11^ and the North American Society for Pediatric Gastroenterology, Hepatology, and Nutrition (NASPGHAN) ^14^ published independent guidelines for the screening, diagnosis, and management of pediatric NAFLD.

NASPGHAN recommends screening for obese children beginning at ages 9 to 11 years as well as overweight children with additional risk factors, including insulin resistance, dyslipidemia, or a family history of NAFLD or cryptogenic cirrhosis. Screening consists of serum alanine aminotransferase (ALT) measurement, using the upper limit of normal (ULN) of 22 U/L for girls and 26 U/L for boys. Patients with an ALT value persistently greater than twice the ULN for at least three months should undergo further evaluation and work-up ^14^. These recommendations are based on expert opinion, whereas AASLD Guidelines do not recommend such screening in adults or children; this is due to a lack of established evidence related to current benefits associated with screening, particularly in the current environment where pharmacologic interventions remain unapproved ^11^. The guidelines purport that since lifestyle modification aimed at weight loss, improved diet, and exercise remains the standard recommendation for all overweight children and adults, a diagnosis of NAFLD will not alter management. This essentially calls into question the utility of screening in the absence of alternative treatment. However, clinicians should consider that confirmation of an existing comorbidity, such as NAFLD, in pediatric obesity may have a role in improving adherence to lifestyle modifications.

In 2015, the European Society for Pediatric Gastroenterology, Hepatology and Nutrition (ESPGHAN) Hepatology Committee published a position paper regarding the role of liver biopsy ^35^. They stated that liver biopsy is required for a definitive diagnosis of NAFLD or NASH but is not proposed in screening. In agreement with AASLD pediatric guidelines, liver biopsy should be performed to exclude other diseases, if advanced disease is suspected, before pharmacologic or surgical treatment, and in clinical research trials ^35^.

## Monitoring

Although percutaneous liver biopsy remains the clinical and research standard for the diagnosis of NASH and for assessing histologic improvement during treatment, several imaging modalities have demonstrated promise as surrogate markers of certain features relating to histologic improvement.

### Vibration-controlled transient elastography

Vibration-controlled transient elastography (VCTE) is an ultrasound-based modality that measures liver stiffness as an estimate of fibrosis. This technique is widely available at point of care, requires minimal training, and provides rapid and reproducible results. VCTE has demonstrated sufficient accuracy for predicting the presence and stability of fibrosis in children with biopsy-proven NAFLD and other chronic liver diseases ^36^.

### Shear wave elastography

Shear wave elastography (SWE) is another ultrasound-based technique that can be used with a conventional ultrasound machine, thereby allowing elastography and anatomical evaluation to be performed in the same sitting. Small cohort studies suggest that SWE is reasonably accurate in distinguishing high- from low-grade fibrosis with cutoff values similar to those reported for adults. This technique requires further validation ^37^.

### Acoustic radiation force impulse

Acoustic radiation force impulse (ARFI) is an ultrasound-based technique that estimates liver fibrosis by assessing tissue stiffness through the evaluation of wave-propagation speed. An ARFI cutoff value of less than 2 m/s reportedly can distinguish between mild and severe fibrosis with 100% sensitivity in children ^15^. This surrogate requires further validation with larger sample sizes at independent sites.

### Magnetic resonance imaging

Although magnetic resonance imaging (MRI) has not been adequately validated as a surrogate marker of histologic improvement in children with NAFLD, emerging data are promising ^38– 40^. Utility appears to be optimal for patients with the highest and lowest degrees of steatosis, and the diagnostic accuracy regarding the mild to moderate range of HF content needs to be improved ^38^. There are insufficient data to relate changes in MRI fat fraction to changes in important NASH features, including inflammation and cell injury. MRI-estimated steatosis may be obfuscated by fibrosis stage. Magnetic resonance elastography offers a potential approach and has been reported to detect advanced fibrosis in pediatric patients, including severely obese patients, with an accuracy of 90% or more ^41^. Barriers to routine use include cost, lack of universal availability, and lack of child cooperation.

## Prevention

Maintaining a healthy lifestyle is the cornerstone of NAFLD prevention. Pediatric health-care providers must regularly assess BMI z-scores. This assessment will allow for an allotment of time during routine health-care visits to discuss appropriate diet, exercise, and family involvement in maintaining weight within an appropriate range. This often requires that obstacles to implementing advice be identified with the help of other ancillary providers, including dietitians, social workers, exercise physiologists, psychologists, and educators.

As previously mentioned, growing evidence suggests that perinatal factors and aspects of the maternal–infant dyad play a contributory role in the development of obesity and the metabolic syndrome, including its hepatic component, NAFLD ^32^. These potentially modifiable risk factors may present an opportunity for early disease prevention. Controlling antepartum hyperglycemia, preventing gestational diabetes, and avoiding excessive weight gain during pregnancy may lead to a decrease in childhood obesity and NAFLD. The promotion of prolonged breastfeeding may also provide long-term benefit for both mother and child ^32^. Further studies are needed to evaluate the long-term benefit of such interventions.

## Management

End-points for treatment studies vary substantially. Commonly pursued and easily measured goals are a decrease in HF fraction and a decrease in or resolution of inflammation and cell injury (that is, NASH) or fibrosis, which are difficult to measure without liver biopsy. This is more difficult in pediatric populations in particular. A sustained decrease in serum ALT from baseline (generally greater than or equal to 50%) is commonly used as a surrogate marker for earlier stages studies relating to histologic improvement and response to treatment ^42, 43^. Fibrosis measures currently accepted by the US Food and Drug Administration (FDA) require histologic assessment obtained via liver biopsy, which is not always feasible in pediatric studies, especially in those controlled with a placebo arm. In children, NAFLD comorbidities (insulin resistance, dyslipidemia, and hypertension) are also important comorbidities to treat in order to improve future outcomes.

### Lifestyle intervention

Lifestyle modification, encompassing dietary modifications and a focus on increased physical activity, constitutes first-line treatment for NAFLD ^11, 14^. Hypocaloric, low-carbohydrate diets and low-fat diets provide similar reductions in intrahepatic fat content as long as weight loss is achieved ^44, 45^. In a randomized clinical study of adolescent boys with NAFLD, a home-delivered diet that was low in free sugars resulted in significant improvement (as assessed by MRI) in hepatic steatosis compared with usual diet ^46^.

Though proven effective, this treatment modality has inherent barriers. As patients with NAFLD often do not exhibit physical symptoms, they frequently lack the motivation required to alter contributory habits and fail to implement prescribed lifestyle changes. Studies confirm that long-term efficacy of diet and lifestyle management in decelerating weight gain has limited success because of inadequate compliance ^47, 48^. Barriers to behavioral change may relate to socioeconomic barriers, including access to affordable healthy food and safe recreational areas and culture-specific beliefs relating health to increased weight.

### Bariatric surgery

Given the inherent difficulties of sustained lifestyle modification, alternative bariatric surgical approaches, including Roux-en-Y gastric bypass (RYGB), laparoscopic adjustable gastric banding, and laparoscopic sleeve gastrectomy (LSG), have been explored, although data are limited to adolescents.

In 2015, the ESPGHAN Hepatology Committee published a position paper regarding the role of bariatric intervention in severely obese children and adolescents with or without NASH ^49^. The authors concluded that bariatric intervention in pediatric patients with morbid obesity results in sustained and clinically significant weight loss and improved NASH but has the potential for serious complications. Therefore, current data are not sufficient to recommend widespread use for weight loss in adolescents without other major comorbidities. As long as appropriate long-term follow-up is provided, RYGB may be considered an effective option for adolescents with extreme obesity associated with other concerning morbidities ^49^.

Manco *et al*. conducted a prospective, multicenter observational study and examined outcomes among obese adolescents with biopsy-proven NAFLD who underwent LSG ^50^. One year after intervention, 20 patients who underwent LSG lost 21.5% of their baseline body weight compared with patients who underwent lifestyle intervention with a decrease from baseline of 1.7%. Six of the six surgical subjects with fibrosis stage 2 were reported to have fibrosis resolution. The authors reported significant improvements in hypertension, hyperlipidemia, and OSA in those undergoing LSG ^50^.

### Pharmacotherapy

At present, there are no FDA-approved pharmacologic agents for the treatment of NAFLD in children or adults. Evidence suggests that d-alpha tocopherol provided at 800 IU orally daily significantly resolves NASH in some patients (both children and adults) with biopsy-proven NASH. Long-term safety remains to be established ^42^. Other proposed therapies include probiotics, insulin sensitizers, and anti-inflammatory, lipid-lowering, and anti-fibrotic agents. Current data are insufficient to support the use of these other agents in pediatric patients. A composite of completed and current pediatric treatment trials is listed in Table 1 and Table 2.

**Table 1.** Current pharmacologic phase 2/3 clinical trials for the treatment of pediatric nonalcoholic fatty liver disease.

	Phase and ClinicalTrials.gov identifier	Category	Proposed mechanism of action
Curcumin	Phase 2 NCT04109742	Antioxidant	Decrease inflammation
Empaglifozin	Phase 2 NCT03867487	Sodium-glucose co-transporter 2 (SGLT2) inhibitor	Decrease insulin resistance
Losartan	Phase 2 NCT03467217	Angiotensin II receptor blocker	Reduces plasminogen activator inhibitor-1 (PAI-1) production and improves insulin sensitivity
IMM-124E	Phase 2 NCT03042767	A bovine colostrum enriched with anti-lipopolysaccharide (LPS) antibodies	Reduces transfer of LPS from gut; anti-inflammatory
Elafibranor	Phase 2 NCT03883607	Peroxisome proliferator- activated receptor (PPAR) α/δ agonist	Improves insulin sensitivity and lipid metabolism and reduces inflammation

**Table 2.** Completed pharmacologic clinical trials for the treatment of pediatric nonalcoholic fatty liver disease.

	Category (mechanism of action)	Recommendation
Metformin ^42^	Insulin sensitizer	Not recommended for treatment of nonalcoholic steatohepatitis (NASH) at dose studied. Consider for management of insulin-resistant patients with nonalcoholic fatty liver disease.
Vitamin E ^42^	Antioxidant	Effective in children with biopsy-proven NASH without diabetes or cirrhosis
Cysteamine bitartrate ^52^	Antioxidant	Not currently recommended for treatment.

## Limitations

One of the primary challenges in pediatric NAFLD has been the variability in clinical trial designs with relatively small sample sizes, heterogeneity of populations, and length of treatments. The absence of validated or accepted non-invasive markers to assess for disease diagnosis or response remains a significant barrier to clinical trial designs and monitoring. Treatment end-points vary across studies. ALT and ultrasound are frequently used as primary outcome surrogates in lieu of liver biopsy given the mandates for trial participation benefits related to clinical research in minors ^51^. Though widely available, these surrogates have not demonstrated sufficient relation with histology. Continued development of age-validated non-invasive biomarkers and imaging will facilitate future study conduct and monitoring of therapeutic response.

## Conclusions

Pediatric NAFLD has emerged as the leading cause of chronic liver disease in children and adolescents worldwide. The pathogenesis of early-onset NAFLD is secondary to a complex interplay involving distinct genetic, metabolic, environmental, and microbiological factors which may begin *in utero*. Lifestyle modifications involving physical activity and dietary changes remain the only practical therapeutic intervention now available, albeit long-term efficacy is limited by poor adherence. Advancements in non-invasive biomarkers and imaging techniques offer promise in assessing the presence and changes in steatosis and fibrosis. Further validation will facilitate longitudinal, natural history, and therapeutic efficacy studies.
